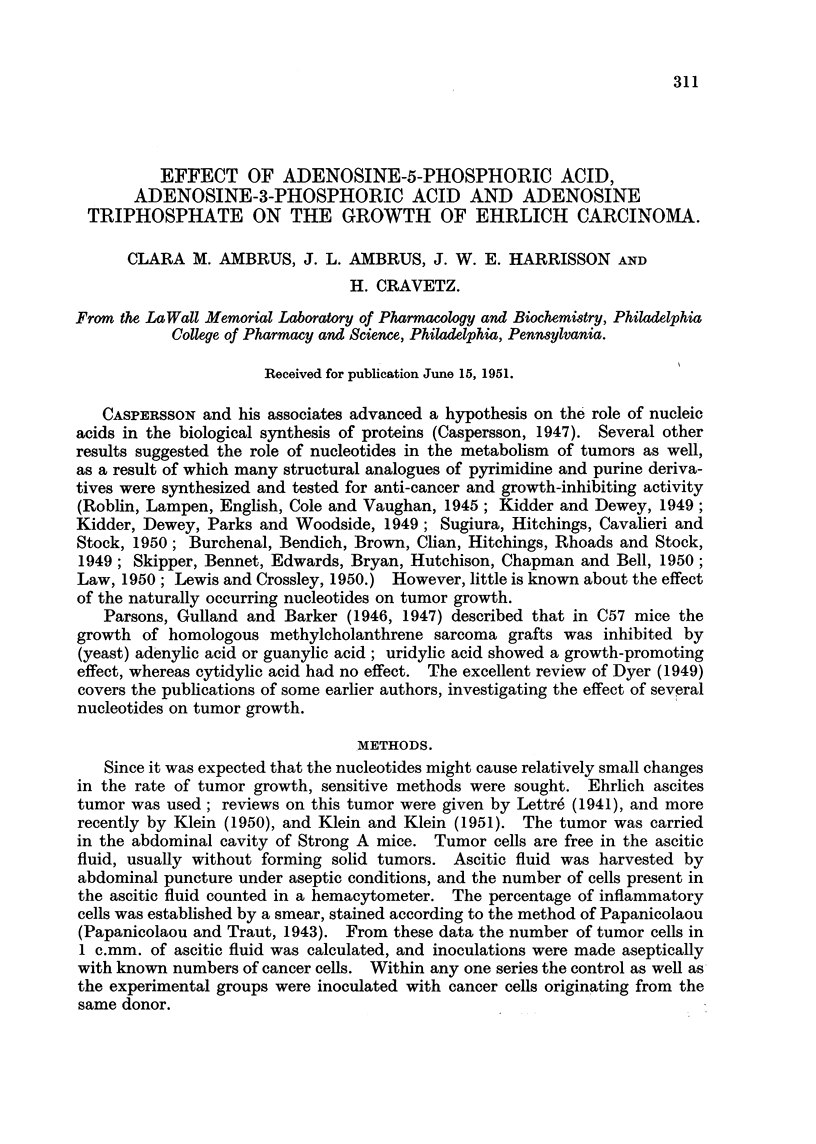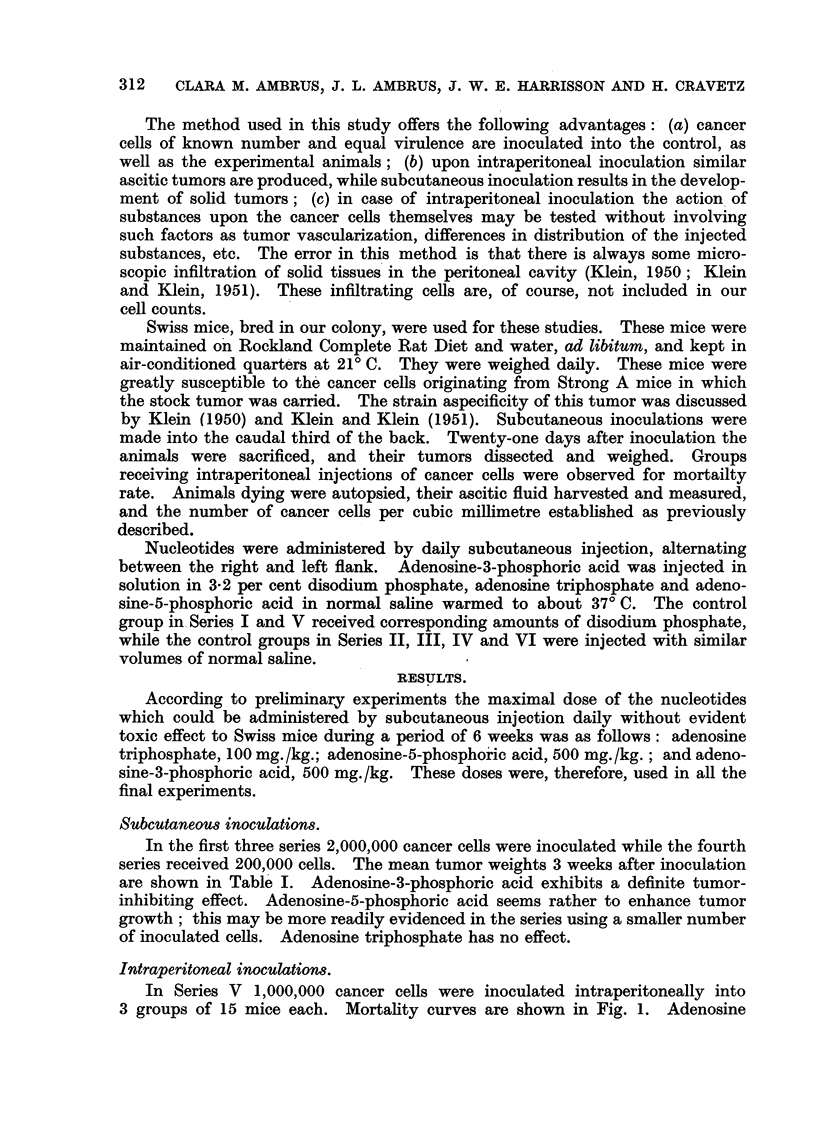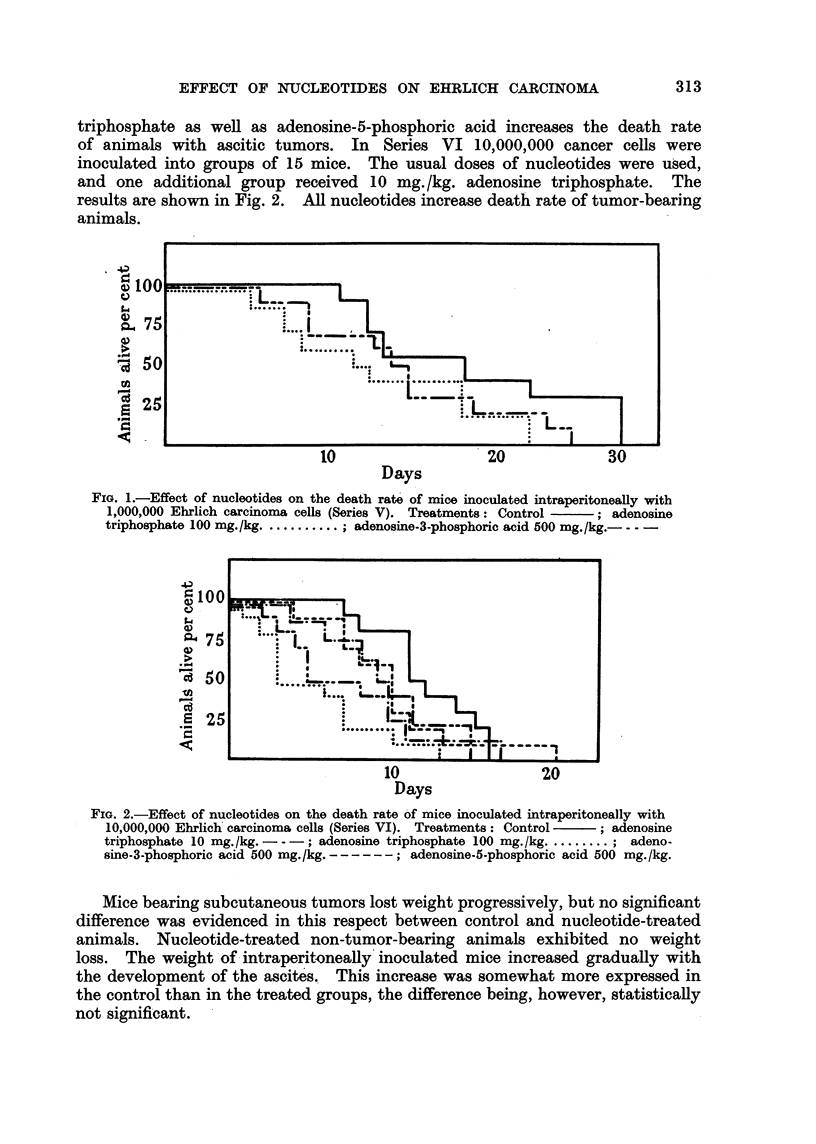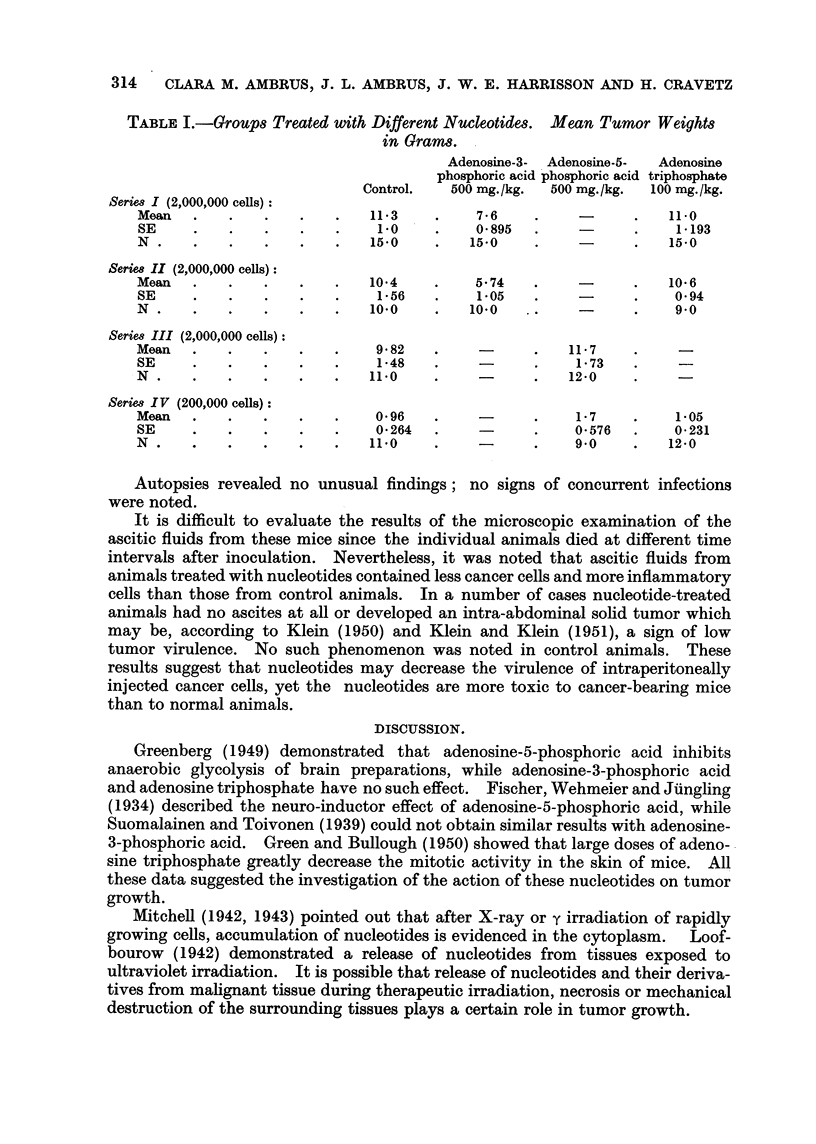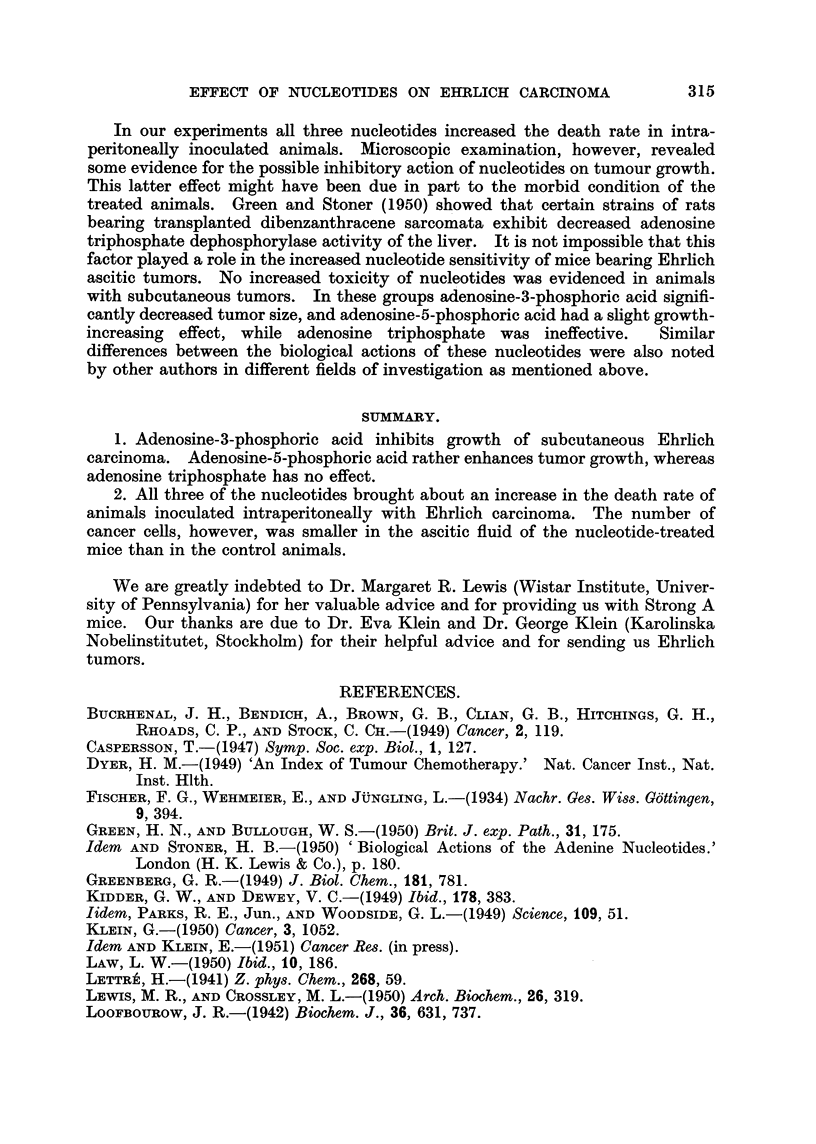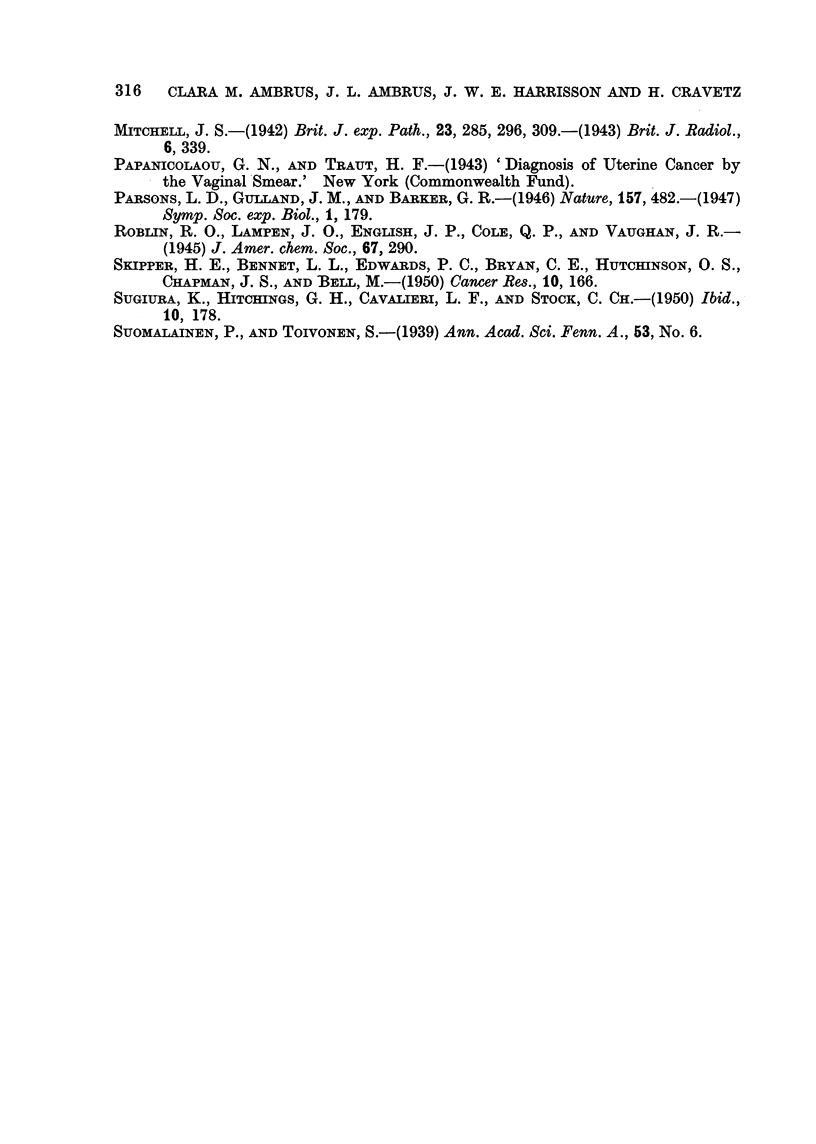# Effect of Adenosine-5-Phosphoric Acid, Adenosine-3-Phosphoric Acid and Adenosine Triphosphate on the Growth of Ehrlich Carcinoma

**DOI:** 10.1038/bjc.1951.32

**Published:** 1951-09

**Authors:** Clara M. Ambrus, J. L. Ambrus, J. W. E. Harrisson, H. Cravetz


### EFFECT OF ADENOSINE-5-PHOSPHORIC ACID, ADENOSINE-3-PHOSPHORIC ACID AND ADENOSINE TRIPHOSPHATE ON THE GROWTH OF EHRLICH CARCINOMA.

CLARA M. AMBRUS, J. L. AMBRUS, J. W. E. HARRISSON AND H. CRAVETZ.

*From the LaWall Memorial Laboratory of Pharmacology and Biochemistry, Philadelphia College of Pharmacy and Science, Philadelphia, Pennsylvania.*

Received for publication June 15, 1951.

CASPERSSON and his associates advanced a hypothesis on the role of nucleic acids in the biological synthesis of proteins (Caspersson, 1947). Several other results suggested the role of nucleotides in the metabolism of tumors as well, as a result of which many structural analogues of pyrimidine and purine derivatives were synthesized and tested for anti-cancer and growth-inhibiting activity (Roblin, Lampen, English, Cole and Vaughan, 1945 ; Kidder and Dewey, 1949 ; Kidder, Dewey, Parks and Woodside, 1949 ; Sugiura, Hitchings, Cavalieri and Stock, 1950 ; Burchenal, Bendich, Brown, Clian, Hitchings, Rhoads and Stock, 1949 ; Skipper, Bennet, Edwards, Bryan, Hutchison, Chapman and Bell, 1950 ; Law, 1950 ; Lewis and Crossley, 1950.) However, little is known about the effect of the naturally occurring nucleotides on tumor growth.

Parsons, Gulland and Barker (1946, 1947) described that in C57 mice the growth of homologous methylcholanthrene sarcoma grafts was inhibited by (yeast) adenylic acid or guanylic acid ; uridylic acid showed a growth-promoting effect, whereas cytidylic acid had no effect. The excellent review of Dyer (1949) covers the publications of some earlier authors, investigating the effect of several nucleotides on tumor growth.

### METHODS.

Since it was expected that the nucleotides might cause relatively small changes in the rate of tumor growth, sensitive methods were sought. Ehrlich ascites tumor was used ; reviews on this tumor were given by Lettré (1941), and more recently by Klein (1950), and Klein and Klein (1951). The tumor was carried in the abdominal cavity of Strong A mice. Tumor cells are free in the ascitic fluid, usually without forming solid tumors. Ascitic fluid was harvested by abdominal puncture under aseptic conditions, and the number of cells present in the ascitic fluid counted in a hemacytometer. The percentage of inflammatory cells was established by a smear, stained according to the method of Papanicolaou (Papanicolaou and Traut, 1943). From these data the number of tumor cells in 1 c.mm. of ascitic fluid was calculated, and inoculations were made aseptically with known numbers of cancer cells. Within any one series the control as well as the experimental groups were inoculated with cancer cells originating from the same donor.

The method used in this study offers the following advantages: (*a*) cancer cells of known number and equal virulence are inoculated into the control, as well as the experimental animals; (*b*) upon intraperitoneal inoculation similar ascitic tumors are produced, while subcutaneous inoculation results in the development of solid tumors; (*c*) in case of intraperitoneal inoculation the action of substances upon the cancer cells themselves may be tested without involving such factors as tumor vascularization, differences in distribution of the injected substances, etc. The error in this method is that there is always some microscopic infiltration of solid tissues in the peritoneal cavity (Klein, 1950; Klein and Klein, 1951). These infiltrating cells are, of course, not included in our cell counts.

Swiss mice, bred in our colony, were used for these studies. These mice were maintained on Rockland Complete Rat Diet and water, *ad libitum*, and kept in air-conditioned quarters at 21° C. They were weighed daily. These mice were greatly susceptible to the cancer cells originating from Strong A mice in which the stock tumor was carried. The strain aspecificity of this tumor was discussed by Klein (1950) and Klein and Klein (1951). Subcutaneous inoculations were made into the caudal third of the back. Twenty-one days after inoculation the animals were sacrificed, and their tumors dissected and weighed. Groups receiving intraperitoneal injections of cancer cells were observed for mortailty rate. Animals dying were autopsied, their ascitic fluid harvested and measured, and the number of cancer cells per cubic millimetre established as previously described.

Nucleotides were administered by daily subcutaneous injection, alternating between the right and left flank. Adenosine-3-phosphoric acid was injected in solution in 3·2 per cent disodium phosphate, adenosine triphosphate and adenosine-5-phosphoric acid in normal saline warmed to about 37° C. The control group in Series I and V received corresponding amounts of disodium phosphate, while the control groups in Series II, III, IV and VI were injected with similar volumes of normal saline.

### RESULTS.

According to preliminary experiments the maximal dose of the nucleotides which could be administered by subcutaneous injection daily without evident toxic effect to Swiss mice during a period of 6 weeks was as follows: adenosine triphosphate, 100 mg./kg.; adenosine-5-phosphoric acid, 500 mg./kg.; and adenosine-3-phosphoric acid, 500 mg./kg. These doses were, therefore, used in all the final experiments.

### *Subcutaneous inoculations.*

In the first three series 2,000,000 cancer cells were inoculated while the fourth series received 200,000 cells. The mean tumor weights 3 weeks after inoculation are shown in Table I. Adenosine-3-phosphoric acid exhibits a definite tumor-inhibiting effect. Adenosine-5-phosphoric acid seems rather to enhance tumor growth; this may be more readily evidenced in the series using a smaller number of inoculated cells. Adenosine triphosphate has no effect.

### *Intraperitoneal inoculations.*

In Series V 1,000,000 cancer cells were inoculated intraperitoneally into 3 groups of 15 mice each. Mortality curves are shown in Fig. 1. Adenosine triphosphate as well as adenosine-5-phosphoric acid increases the death rate of animals with ascitic tumors. In Series VI 10,000,000 cancer cells were inoculated into groups of 15 mice. The usual doses of nucleotides were used, and one additional group received 10 mg./kg. adenosine triphosphate. The results are shown in Fig. 2. All nucleotides increase death rate of tumor-bearing animals.

FIG. 1.—Effect of nucleotides on the death rate of mice inoculated intraperitoneally with 1,000,000 Ehrlich carcinoma cells (Series V). Treatments: Control ———; adenosine triphosphate 100 mg./kg. ..........; adenosine-3-phosphoric acid 500 mg./kg.— - - —

FIG. 2.—Effect of nucleotides on the death rate of mice inoculated intraperitoneally with 10,000,000 Ehrlich carcinoma cells (Series VI). Treatments: Control ———; adenosine triphosphate 10 mg./kg. — - —; adenosine triphosphate 100 mg./kg. .........; adenosine-3-phosphoric acid 500 mg./kg. - - - - - - -; adenosine-5-phosphoric acid 500 mg./kg.

Mice bearing subcutaneous tumors lost weight progressively, but no significant difference was evidenced in this respect between control and nucleotide-treated animals. Nucleotide-treated non-tumor-bearing animals exhibited no weight loss. The weight of intraperitoneally inoculated mice increased gradually with the development of the ascites. This increase was somewhat more expressed in the control than in the treated groups, the difference being, however, statistically not significant.

TABLE I.—*Groups Treated with Different Nucleotides. Mean Tumor Weights in Grams.*

| | Control. | Adenosine-3-phosphoric acid 500 mg./kg. | Adenosine-5-phosphoric acid 500 mg./kg. | Adenosine triphosphate 100 mg./kg. |
|---|---|---|---|---|
| *Series I* (2,000,000 cells) : | | | | |
| Mean | 11·3 | 7·6 | — | 11·0 |
| SE | 1·0 | 0·895 | — | 1·193 |
| N | 15·0 | 15·0 | — | 15·0 |
| *Series II* (2,000,000 cells) : | | | | |
| Mean | 10·4 | 5·74 | — | 10·6 |
| SE | 1·56 | 1·05 | — | 0·94 |
| N | 10·0 | 10·0 | — | 9·0 |
| *Series III* (2,000,000 cells) : | | | | |
| Mean | 9·82 | — | 11·7 | — |
| SE | 1·48 | — | 1·73 | — |
| N | 11·0 | — | 12·0 | — |
| *Series IV* (200,000 cells) : | | | | |
| Mean | 0·96 | — | 1·7 | 1·05 |
| SE | 0·264 | — | 0·576 | 0·231 |
| N | 11·0 | — | 9·0 | 12·0 |

Autopsies revealed no unusual findings ; no signs of concurrent infections were noted.

It is difficult to evaluate the results of the microscopic examination of the ascitic fluids from these mice since the individual animals died at different time intervals after inoculation. Nevertheless, it was noted that ascitic fluids from animals treated with nucleotides contained less cancer cells and more inflammatory cells than those from control animals. In a number of cases nucleotide-treated animals had no ascites at all or developed an intra-abdominal solid tumor which may be, according to Klein (1950) and Klein and Klein (1951), a sign of low tumor virulence. No such phenomenon was noted in control animals. These results suggest that nucleotides may decrease the virulence of intraperitoneally injected cancer cells, yet the nucleotides are more toxic to cancer-bearing mice than to normal animals.

### DISCUSSION.

Greenberg (1949) demonstrated that adenosine-5-phosphoric acid inhibits anaerobic glycolysis of brain preparations, while adenosine-3-phosphoric acid and adenosine triphosphate have no such effect. Fischer, Wehmeier and Jüngling (1934) described the neuro-inductor effect of adenosine-5-phosphoric acid, while Suomalainen and Toivonen (1939) could not obtain similar results with adenosine-3-phosphoric acid. Green and Bullough (1950) showed that large doses of adenosine triphosphate greatly decrease the mitotic activity in the skin of mice. All these data suggested the investigation of the action of these nucleotides on tumor growth.

Mitchell (1942, 1943) pointed out that after X-ray or $\gamma$ irradiation of rapidly growing cells, accumulation of nucleotides is evidenced in the cytoplasm. Loofbourow (1942) demonstrated a release of nucleotides from tissues exposed to ultraviolet irradiation. It is possible that release of nucleotides and their derivatives from malignant tissue during therapeutic irradiation, necrosis or mechanical destruction of the surrounding tissues plays a certain role in tumor growth.

In our experiments all three nucleotides increased the death rate in intraperitoneally inoculated animals. Microscopic examination, however, revealed some evidence for the possible inhibitory action of nucleotides on tumour growth. This latter effect might have been due in part to the morbid condition of the treated animals. Green and Stoner (1950) showed that certain strains of rats bearing transplanted dibenzanthracene sarcomata exhibit decreased adenosine triphosphate dephosphorylase activity of the liver. It is not impossible that this factor played a role in the increased nucleotide sensitivity of mice bearing Ehrlich ascitic tumors. No increased toxicity of nucleotides was evidenced in animals with subcutaneous tumors. In these groups adenosine-3-phosphoric acid significantly decreased tumor size, and adenosine-5-phosphoric acid had a slight growth-increasing effect, while adenosine triphosphate was ineffective. Similar differences between the biological actions of these nucleotides were also noted by other authors in different fields of investigation as mentioned above.

### SUMMARY.

1. Adenosine-3-phosphoric acid inhibits growth of subcutaneous Ehrlich carcinoma. Adenosine-5-phosphoric acid rather enhances tumor growth, whereas adenosine triphosphate has no effect.

2. All three of the nucleotides brought about an increase in the death rate of animals inoculated intraperitoneally with Ehrlich carcinoma. The number of cancer cells, however, was smaller in the ascitic fluid of the nucleotide-treated mice than in the control animals.

We are greatly indebted to Dr. Margaret R. Lewis (Wistar Institute, University of Pennsylvania) for her valuable advice and for providing us with Strong A mice. Our thanks are due to Dr. Eva Klein and Dr. George Klein (Karolinska Nobelinstitutet, Stockholm) for their helpful advice and for sending us Ehrlich tumors.

### REFERENCES.

BUCRHENAL, J. H., BENDICH, A., BROWN, G. B., CLIAN, G. B., HITCHINGS, G. H., RHOADS, C. P., AND STOCK, C. CH.—(1949) *Cancer*, **2**, 119.

CASPERSSON, T.—(1947) *Symp. Soc. exp. Biol.*, **1**, 127.

DYER, H. M.—(1949) 'An Index of Tumour Chemotherapy.' Nat. Cancer Inst., Nat. Inst. Hlth.

FISCHER, F. G., WEHMEIER, E., AND JÜNGLING, L.—(1934) *Nachr. Ges. Wiss. Göttingen*, **9**, 394.

GREEN, H. N., AND BULLOUGH, W. S.—(1950) *Brit. J. exp. Path.*, **31**, 175.

*Idem* AND STONER, H. B.—(1950) 'Biological Actions of the Adenine Nucleotides.' London (H. K. Lewis & Co.), p. 180.

GREENBERG, G. R.—(1949) *J. Biol. Chem.*, **181**, 781.

KIDDER, G. W., AND DEWEY, V. C.—(1949) *Ibid.*, **178**, 383.

*Iidem*, PARKS, R. E., Jun., AND WOODSIDE, G. L.—(1949) *Science*, **109**, 51.

KLEIN, G.—(1950) *Cancer*, **3**, 1052.

*Idem* AND KLEIN, E.—(1951) *Cancer Res.* (in press).

LAW, L. W.—(1950) *Ibid.*, **10**, 186.

LETTRÉ, H.—(1941) *Z. phys. Chem.*, **268**, 59.

LEWIS, M. R., AND CROSSLEY, M. L.—(1950) *Arch. Biochem.*, **26**, 319.

LOOFBOUROW, J. R.—(1942) *Biochem. J.*, **36**, 631, 737.

MITCHELL, J. S.—(1942) *Brit. J. exp. Path.*, **23**, 285, 296, 309.—(1943) *Brit. J. Radiol.*, **6**, 339.

PAPANICOLAOU, G. N., AND TRAUT, H. F.—(1943) 'Diagnosis of Uterine Cancer by the Vaginal Smear.' New York (Commonwealth Fund).

PARSONS, L. D., GULLAND, J. M., AND BARKER, G. R.—(1946) *Nature*, **157**, 482.—(1947) *Symp. Soc. exp. Biol.*, **1**, 179.

ROBLIN, R. O., LAMPEN, J. O., ENGLISH, J. P., COLE, Q. P., AND VAUGHAN, J. R.—(1945) *J. Amer. chem. Soc.*, **67**, 290.

SKIPPER, H. E., BENNET, L. L., EDWARDS, P. C., BRYAN, C. E., HUTCHINSON, O. S., CHAPMAN, J. S., AND BELL, M.—(1950) *Cancer Res.*, **10**, 166.

SUGIURA, K., HITCHINGS, G. H., CAVALIERI, L. F., AND STOCK, C. CH.—(1950) *Ibid.*, **10**, 178.

SUOMALAINEN, P., AND TOIVONEN, S.—(1939) *Ann. Acad. Sci. Fenn. A.*, **53**, No. 6.